# Global scoping review of key domains of patient-reported experience of care measures across life stages and healthcare technical areas

**DOI:** 10.1136/bmjopen-2025-103782

**Published:** 2026-01-16

**Authors:** Helen H Habib, Connie Zhang, Kathleen Hill, Lara M E Vaz, Barbara Rawlins, Özge Tunçalp, Moise Muzigaba, Ian Brownwood, Patience A Afulani

**Affiliations:** 1 African Population and Health Research Center, Nairobi, Kenya; 2 University of Ghana School of Public Health, Accra, Ghana; 3 Folio Health, Kansas City, Kansas, USA; 4 JHPIEGO, Washington DC, NW, District of Columbia, USA; 5 Population Reference Bureau, Washington, District of Columbia, USA; 6 USAID, Washington, District of Columbia, USA; 7 Institute of Tropical Medicine, Antwerp, Belgium; 8 Department of Sexual, Reproductive, Maternal, Child, Adolescent and Ageing Health, World Health Organization, Geneva, Switzerland; 9 Health Policy Analysis Pty Ltd, St Leonards, New South Wales, Australia; 10 Obstetrics, Gynecology, and Reproductive Sciences, University of California San Francisco, San Francisco, California, USA

**Keywords:** Quality Improvement, Health Services, Quality in health care

## Abstract

**Abstract:**

**Objectives:**

Patient-reported experience measures (PREMs) are measures of patients’ perceptions of care they receive. PREMs are critical in developing and evaluating programmes that aim to improve patient healthcare experiences and quality of care (QoC) according to patient-defined needs. This review aims to map key domains of PREMs across distinct healthcare technical areas and life stages from globally available literature.

**Design:**

A scoping review adapting Arksey and O’Malley’s framework and Joanna Briggs Institute’s guidelines for the conduct of scoping reviews.

**Data sources:**

Google Scholar, PubMed, WHO, US Academy of Medicine and USAID Momentum.

**Eligibility:**

PREMs literature from electronic repositories of grey and peer-reviewed publications, published in English historically up to September 2023.

**Data extraction and analysis:**

Two lead reviewers with support from the technical working group co-created a review framework of healthcare technical areas, life stages and PREMs domains. We screened eligible articles, prioritising reviews except for technical areas with no reviews, where we then selected individual studies. We charted, analysed and synthesised data from 52 eligible articles.

**Results:**

PREMs literature has recently increased, especially in low-income and middle-income countries (LMICs), although studies in high-income countries (HICs) dominate in proportion (n=38; 73.1%). Out of 52 eligible articles, technical areas with most publications were sexual and reproductive health (n=21; 40.4%) and general outpatient care (n=11; 21.2%). Studies in adulthood (n=24; 46.2%) and from pregnancy and birth to postnatal (n=16; 30.8%) were most represented. PREMs studies reported mostly on communication and rapport (n=33; 63.5%) and respect and dignity (n=42; 80.8%) domains. Nearly a quarter (n=12; 23.1%) of the articles included only validated tools; the rest included a combination of validated and unvalidated measures. Of the tools relating to life stages of babies, younger children and older adults, the majority (n=17; 94.4%) included patient proxies.

**Conclusion:**

PREMs, as an important component of QoC measurement, are increasing across several healthcare technical areas and life stages with commonalities and notable distinctions in measurement domains and tools. Evidence on PREMs largely comes from HICs. Evidence on critical, yet sometimes overlooked domains, highlights key QoC implementation gaps. The adaptation and utilisation of PREMs in programmes, especially in LMICs and under-represented technical areas, present opportunities to close the QoC disparities in those settings. Strategic, concerted efforts towards the harmonisation of PREMs tools across multiple life course stages and technical areas are critically needed in high-level quality improvement efforts.

**STRENGTHS AND LIMITATIONS OF THIS STUDY**

- This review used an inclusive, comprehensive search strategy including peer-reviewed, grey and hand-searched recommended literature.
- This analysis and presentation of evidence in this review were centred around the evidence-based life-course framework.
- There is extensive heterogeneity and overlap of technical areas and life-course stages in several articles.
- This review includes articles published historically up until September 2023. Evidence available after this period is excluded.
- This review includes only articles published in English.

## Introduction

### Background

 Globally, healthcare improvement efforts are increasingly aiming to centre patients and their needs as a quality improvement priority. By employing strategies to improve patient-centred care that includes regular measurement of patients’ experience of care, healthcare programmes are progressively focused on developing initiatives that promote the responsiveness of healthcare services to the needs and preferences of clients.^1 2^ Relatedly, patient experiences, not just clinical or patient-reported outcomes, have been highlighted as critical aspects of quality care that require the monitoring of well-defined and actionable indicators. PREMs have been described as “a measure of a patient’s perception of their personal experience of the healthcare they have received”.^3^ PREMs inherently are an invaluable opportunity to identify and remedy non-clinical, service quality gaps that may otherwise be unidentified and missed from the provider perspective. This is for the simple reason that patients themselves are best able to evaluate and relay their own experiences with healthcare programmes and health systems. Widely accepted domains of quality of care (QoC), which PREMs seek to assess, include communication and rapport, privacy and confidentiality, respect and dignity, and autonomy.^4 5^

PREMs are distinguished from patient-reported outcome measures, which measure the self-reported health status and clinical outcomes of patients.^6^ These include signs and symptoms, functionality and health-related quality of life.^6^ PREMs are also distinguished from client satisfaction measures, which measure the care expectations of the patient relative to the actual care provided. In contrast, PREMs attempt to assess actual experiences that occurred regardless of patient expectations.

There is growing evidence globally on PREMs across several healthcare technical areas, especially in sexual and reproductive healthcare and geriatric care.^7^,^10^ However, it appears this evidence is mostly concentrated in high-income countries (HICs) as classified by the World Bank such as^11^ Australia, North America and Europe, with relatively sparser literature emerging from low-income and middle-income countries (LMICs).^4^ Nonetheless, the concept of PREMs is generally gaining traction in scope and scale as healthcare programmes move towards improving healthcare to be more responsive to patients’ experiences while prioritising patient agency.^2 12^

Concurrently, with this ongoing proliferation, there are challenges with the standardisation of PREMs instruments across technical areas, life stages and geographical contexts. While this may be unsurprising due to both discrete and nuanced differences in the delivery and receipt of care across various technical areas and life stages, there remains a need to address challenges in the identification, collation and harmonisation of PREMs as useful measurement tools across programmes. Considering the diverse range of PREMs instruments across different technical areas and life stages, prioritising approaches to comparability between validated and more newly developed measures is also of importance. This could, relatedly, improve the harmonisation of quality assessments of healthcare programmes across varying contexts. This also potentially reduces data-related burdens for implementers and patients who may be fatigued by redundant tools and instruments.

### Objectives

The aim of this review was to retrieve and describe globally extant literature on patient-reported experience of care domains from an open-ended start period up until 2023. Specifically, the review sought to map PREMs domains from distinct healthcare programme technical areas and across life stages. This review was undertaken under the auspices of the *WHO Life Stage Quality of Care Measurement Technical Working Group* (*LSQM TWG*). The *LSQM TWG* is composed of experts from a range of disciplines and professional backgrounds, including researchers and programme implementers working in the areas of public health, QoC, patient-reported experience of care, equity, monitoring, evaluation, reproductive, maternal, newborn and child health, ageing and primary healthcare. *Task Team 3* (*TT3*), one of three task teams in the *LSQM TWG*, has focused on the development of knowledge and practical products related to the measurement of client and provider experience of care and patient-reported health outcomes. This mapping of domains of interest is an important step in identifying measurement priorities in different technical areas and life-course stages. A scoping review was needed to first identify the relevant PREMs studies to include in the mapping.

## Methods

**Figure 1:**
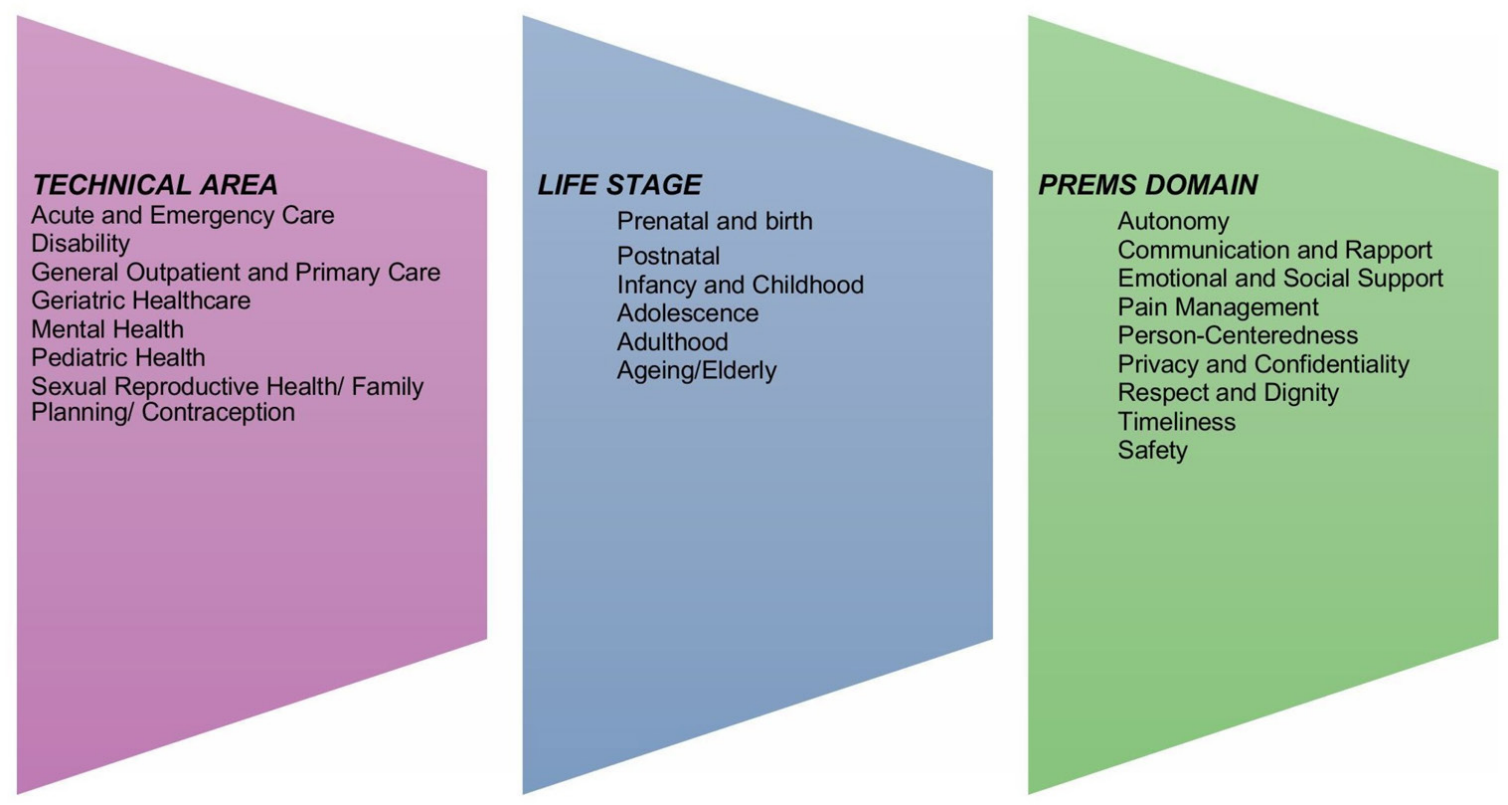
Key domains of patient-reported experience of care measures across life stages and healthcare technical areas.

This review exercise adapted a hybrid of the methodology for conducting a scoping review outlined by the Joanna Briggs Institute^13^ and the scoping review guidelines proffered by Arksey and O’Malley.^14^ This adaptation of both methods was preferred to facilitate the production of a more comprehensive and inclusive exercise by incorporating diverse inputs of technical professionals beyond the two primary reviewers. This exercise therefore observed the recommendations of transparent and consultative approaches at each stage. The process accordingly began with the consultative co-development of a clear and precise protocol to guide the conduct of the review. The protocol detailed the specific research questions to be answered by this review and precise procedures to be followed in its conduct. Additionally, frequent consultations were convened with members of the *LSQM TWG-TT3* for their technical input. Inputs from the TWG included appraisal and refinement of the review protocol, development of review framework and recommendations of relatively obscure sources of literature. We adopted an iterative approach to examine literature and map PREMs domains across life stages. In a collaborative exercise, technical healthcare areas were prioritised in this exercise based on consensus of the *LSQM TWG*. The framework for the mapping of PREMs domains, life stages and healthcare technical areas is presented in figure 1. This framework was adapted at the beginning of this process in reference to existing frameworks, including “*Patient-reported indicators for assessing health system performance”*^4^ and “*Primary health care performance measurement for improvement: framework and indicators”*.^5^ Patients and/or the public were not involved in the design, or conduct, or reporting, or dissemination plans of this research.

### Searching for relevant studies

We searched peer-reviewed and grey reviews of literature from electronic repositories to identify texts focused on PREMs studies and their related measures. We used a combination of grouped medical subject headings related to life stages and healthcare technical domains. We searched Google Scholar specialised search engine to find relevant articles. We also searched peer-reviewed literature databases, including PubMed and Embase. The grey literature sources searched were the websites of US Academy of Medicine, WHO and United States Agency for International Development. Both qualitative and quantitative studies were considered, with a priority focus on reviews published historically up until 2023. This wide scope of included literature was selected to avoid the exclusion of important literature in certain study designs or time periods.

Literature recommendations from members of the *LSQM TWG-TT3* were also searched manually from recommended databases and included based on consensus of the group.

The search strategy is presented in online supplemental Appendix A.

### Eligibility criteria

#### Inclusion

A publication was considered eligible for full-text screening if it met the following criteria:

1. The article or publication reports on or uses PREMs.
2. The article or publication is a review.
3. The article or publication is written in English.
4. The article or publication could be found through an open search period historically up to September 2023.

#### Exclusion

We did not include literature on individual studies unless we could not locate any reviews for a particular PREMs domain and/or healthcare technical area. In those scenarios, individual studies that were considered eligible were those that aimed to report on a PREMs instrument for use in a healthcare technical area or life-course domain. This excluded protocols and metrics development studies. Only patient-reported or caregiver-proxy *and* patient-reported experiences were included as part of this review, even though some studies additionally included healthcare provider proxies. Publications that were exclusively comprised of clinician and healthcare provider-reported experiences were excluded from this review.

### Study selection

We uploaded studies yielded from our database searches into Covidence software. After removing duplicates, the two primary authors (CZ and HHH) independently screened titles and abstracts. Those articles not considered directly applicable to answering our specific review questions were excluded at this stage. The remainder underwent full-text screening by the two primary authors; those determined to not meet the review criteria were excluded. Articles that met the inclusion criteria underwent full-text data extraction and charting. Any discrepancies during the screening stages were resolved by the two primary reviewers through consensus following a joint review of the article.

### Data charting

The studies meeting the inclusion criteria of this review were exported from Covidence into a custom-designed Microsoft Excel spreadsheet for charting by the two primary reviewers. Charted data included the year of publication, type of review and the countries of the individual studies in each review (when available). We also extracted information on the aims and objectives of each article. To answer our research questions, we charted specific data on PREMs tools and instruments based on our classification in the indicator framework depicted in figure 1. This included details on the life stage the study focused on, the technical area of interest and the PREMs domain(s) covered.

### Analysis and synthesis

We carried out a basic descriptive analysis of the background details of the included articles to assess the temporal and geographical distribution of the studies. This analysis also assessed the most common review designs and types. We analysed the frequency of PREMs studies conducted for each of the healthcare technical areas and life stages per PREMs domain. We proceeded to conduct a narrative synthesis of the charted articles, describing the characteristics of the articles according to framework components. This included synthesising PREMs domains, healthcare technical areas and life stages each article assessed. For life stages, we identified PREMs domains common to most life stages and noted those unique to specific life stages. In the same manner, we analysed and synthesised PREMs domains across each healthcare technical area, highlighting domains unique to each technical area and those that were more cross-cutting in representation. We consulted with the members of *LSQM TWG-TT3* during the thematic synthesis for input on identified themes, gaps and related recommendations.

## Results

### Characteristics of included studies

**Figure 2:**
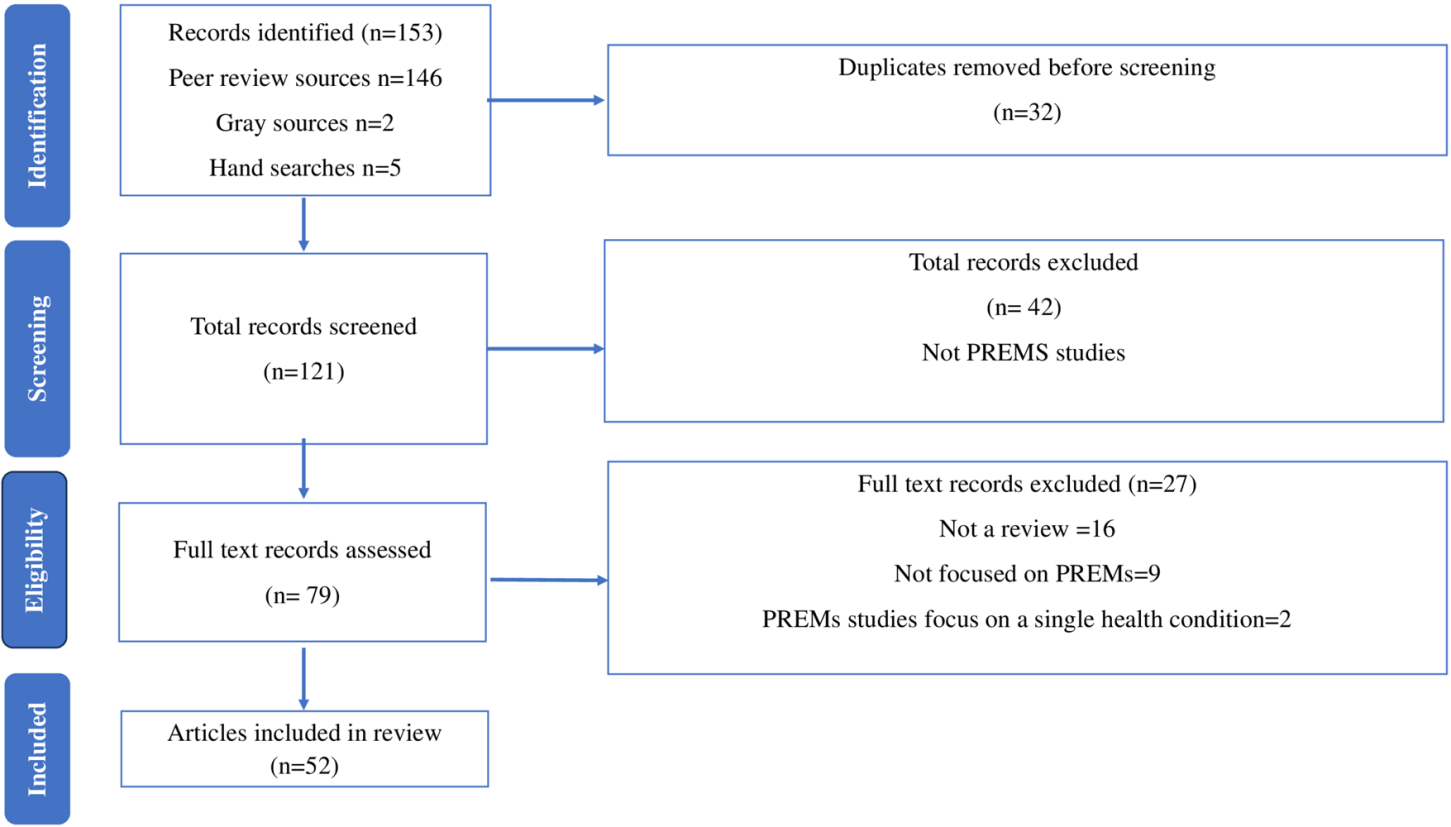
Preferred Reporting Items for Systematic Reviews and Meta-Analyses flow diagram. PREMs, patient-reported experience measures.

In the context of this study, ‘articles’ refers to the manuscripts we retrieved from the literature sources while ‘individual studies’ refers to the constituent studies in each article, keeping in mind that we mainly focused on review articles which by design aggregate several studies. Our initial electronic database and manual hand searching yielded a total output of 153 articles. After removing duplicates, we were left with 121 unique articles. These articles underwent a preliminary title and abstract screening by each of the primary reviewers, resulting in the exclusion of 42 articles. Articles that passed the title and abstract screening stage underwent a full-text screening, which further excluded 27 articles. After full-text screening, 52 articles were identified as eligible for inclusion in this review and were retained for extraction. This comprised 50 review articles, 1 research note and 1 single study manuscript. All the included articles applied to at least one PREMs domain in any of the healthcare technical areas and/or life stages in our framework. The results of the searches and screening are illustrated visually in the Preferred Reporting Items for Systematic Reviews and Meta-Analyses flow diagram as in figure 2.

**Figure 3:**
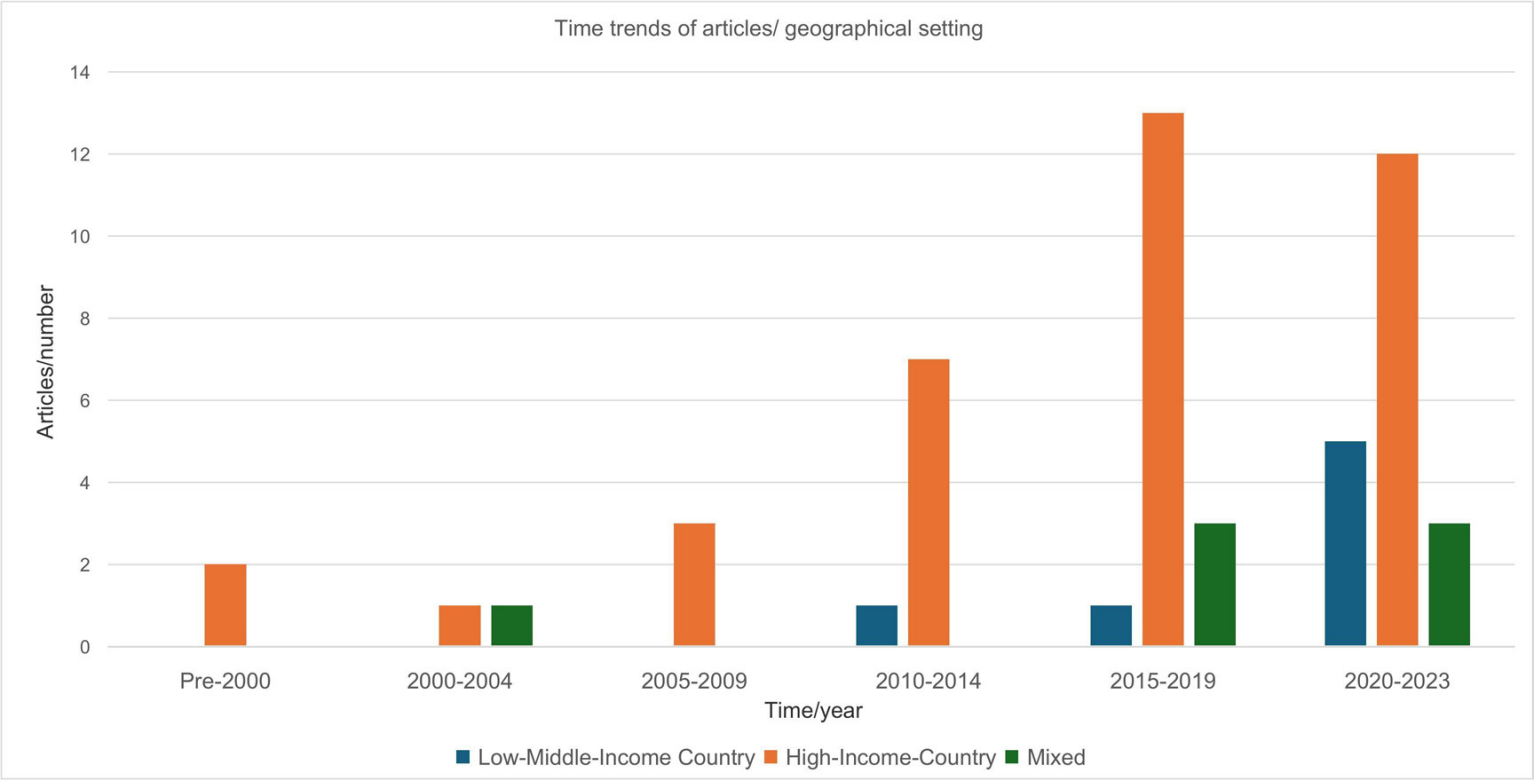
Publication date and category of country for included articles.

Articles included in this review were published between 1997 and 2023, with only two (3.8%) articles published prior to 2000. The majority of articles (n=44; 84.6%) were published in the past decade. Over two-thirds (n=10; 71.4%) of the 14 studies focusing on LMICs were published in the past 5 years. Studies conducted in HICs had a relatively more even temporal distribution over time. Most of the review articles (n=38; 73.1%) reported on individual studies that were conducted in HICs, including Australia, Canada, Europe, the UK and the USA. Almost two-thirds (n=6; 75%) of the studies conducted in LMICs were carried out in sub-Saharan Africa. Figure 3 illustrates the trends of publication dates and category of country distribution of articles in this review.

Over half (n=30; 57.7%) of the articles in our review included studies from both grey and peer-reviewed sources of literature. The rest of the included articles only included individual studies from peer-reviewed literature sources. Nearly three quarters (n=38; 73.1%) of the articles were systematic reviews, while less than a tenth (n=4; 7.7%) were scoping reviews. The rest of the included articles were in the form of other designs, including narrative reviews and general literature reviews. Most of the included reviews (n=39; 75.0%) included individual studies with both quantitative and qualitative designs. Nearly a fifth (n=9; 17.3%) of the studies comprised only qualitative individual studies. About one-third (n=17; 32.7%) of the included articles comprised individual studies that only included patient views. The rest of the articles (n=35; 67.3%) included studies where participants included both the patient and a proxy respondent. Proxy respondents comprised caregivers (eg, parents of children and adult children of the elderly). Among studies involving proxy respondents, those that focused on the life stages of babies, younger children and the elderly generally (n=17; 94.4%) included patient proxies in addition to the direct patient. In other life stages, 61.9% of PREMs studies included both patient participants and their caregivers and health providers

### PREMs domains across healthcare technical areas and life stages

Based on our initial framework, the healthcare technical areas with the most PREMs articles represented in this review were sexual and reproductive health (SRH) (n=21; 40.4%) and general outpatient care (n=11; 21.2%). PREMs related to acute/emergency care (n=1; 1.9%) and disability care (n=2; 3.8%) were the least represented technical areas. Other technical areas in our review with low representation included mental health (n=3; 5.8%) and paediatric care (n=7; 13.5%).

Almost half of the included articles focused on the adulthood life stage (n=24; 46.2%). Most articles operationalised adulthood as 18 years and older. This definition generally overlapped with elderly populations, although some articles specifically highlighted elderly or ageing populations (n=11; 21.2%). This overlap of life stages was similarly present in the pregnancy and birth as well as postnatal literature. The representation of other life stages in the included literature included pregnancy and birth (n=9; 17.3%), postnatal (n=7; 13.5%), infancy and childhood (n=7; 13.5%) and adolescence (n=8; 15.4%).

Table 1 presents PREMs studies mapped by healthcare technical area and life stage. Given the nature of comprehensive systematic reviews, multiple healthcare technical areas (eg, outpatient health and emergency care) were often synthesised within a single publication by the shared domains they covered. Accordingly, some of the individual reviews overlapped our framework’s classifications of healthcare technical areas and life stages. Due to these overlaps, the total count of articles in table 1 exceeds 52 studies.

**Table 1:** Content of reviewed articles reporting on patient-reported experience measures by healthcare technical areas and life stages

	Life stage					
Technical healthcare area	Pregnancy and birth	Postnatal	Infancy and childhood	Adolescence	Adulthood	Ageing/ elderly
Acute/emergency care					1 review	
Geriatric health					2 reviews	8 reviews
Disability					2 reviews	1 review
General outpatient and primary healthcare				1 review	9 reviews 1 research note	2 reviews
Mental health		1 single study	1 single study		2 reviews	
Paediatric health	2 reviews	3 reviews	6 reviews	3 reviews	1 review	
Sexual and reproductive health and family planning	11 reviews	3 reviews		4 reviews	6 reviews	

Legend (number of reviews): 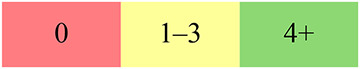

Over one-third of the articles in this review (n=20; 38.5%) did not provide details on the specific PREMs tools and instruments employed in their respective individual studies. Less than a quarter of the included studies (n=13; 23.1%) specified that they only included individual studies using validated PREMs tools and instruments. A quarter (n=14; 26.9%) did not specify whether the tools used in the individual studies were validated or not. The remainder of studies specified that they included studies that used both validated and unvalidated measures. The most frequently cited validated PREMs instruments in the review studies were the *Person-Centered Maternity Care* Scale,^15^,^17^ the *Picker Patient Survey*^10 18 19^ and the *Consumer Assessment of Healthcare Providers and Systems survey*.^1218^,^24^ A list of all referenced PREMs tools in the included articles is presented in online supplemental Appendix B.

### Common domains

Among the PREMs domains in our framework, some were ubiquitous across life stages and in healthcare technical areas. The most commonly occurring PREMs domain was respect and dignity, which appeared in over two-thirds (n=42; 80.8%) of the included studies. Other frequently occurring domains included safety (n=34; 65.4%), communication and rapport (n=33; 63.5%), privacy and confidentiality (n=31; 59.6%) and pain management (n=29; 55.8%). Comparatively less frequently occurring domains included timeliness in service delivery, which appeared in approximately a third (n=18; 34.6%) of the articles in our review. Some studies presented a single composite domain of person-centred care, which is described as “care approaches and practices that see the person as a whole with many levels of needs and goals, with these needs coming from their own personal social determinants of health”,^25^ and encompasses elements of respect, autonomy and timeliness.^12 15 17 26 27^ We included the domain person-centredness to capture these studies.

Domains reported in the included literature are presented in table 2.

**Table 2:** Identified patient-reported experience measures domains (N=52)

Domains	*n*	%	References
Autonomy	22	42.3	47 10 15 17 21 24 27 42 46
Communication and rapport	33	63.5	46 7 15 19 21
Pain management	29	55.8	67 10 16 18 22 24 26 42 44 47 53
Person-centredness	22	42.3	46 10 12 15 17 19 21 27 47 48 50 51 54 57 60 61 66 72 74 75
Privacy and confidentiality	31	59.6	46 7 9 10 15 17 19 21 22 24 27 48 49 52 56 58 60
Respect and dignity	42	80.8	67 9 10 12 15 17 19
Safety	34	65.4	46 10 12 15 17 24 42 46
Timeliness	18	34.6	1012 15 17 23 27 47 49
Social and emotional support	19	36.5	415 21 22 24 27 48 51 53 57 59 61 64 66 70 73

### Distinct domains

The identified domains from our framework revealed distinctions among certain healthcare technical areas. For instance, the domain of pain management appeared universally among studies focused on the healthcare technical areas of paediatrics and acute and emergency care. In comparison, just about half of the studies on general outpatient department explored this domain.

Similarly, all the studies in paediatric care explored the domain of emotional and social support, and over two-thirds (n=6; 75.0%) of the studies in geriatric healthcare also explored this domain. This distinction of domains was also demonstrated along life stages. Communication and rapport were explored in over two-thirds of studies focusing on the stage of pregnancy and birth (n=7; 77.8%) and adolescence (n=7; 87.5%). By comparison, only 18.2% (n=2) of studies focused on ageing/elderly populations explored this domain. In the same vein, emotional and social support was examined in almost all the studies focusing on the postnatal stage (n=6; 85.7%) and infancy/childhood (n=6; 85.7%). Privacy and confidentiality were also concentrated among adolescent studies with almost all articles focused on that life stage assessing that domain (n=7; 87.5%). Table 3 presents the distribution of PREMs domains across the life stages.

**Table 3:** Matrix of patient-reported experience measures domains by life stages

	Pregnancy and birth (n=9) (%)	Postnatal(n=7) (%)	Infancy/childhood(n=7) (%)	Adolescence(n=8) (%)	Adulthood(n=23) (%)	Ageing/ elderly(n=11) (%)
Dignity and respect	66.70	42.90	42.90	87.50	82.60	18.20
Safety	66.70	28.60	42.90	62.50	78.30	72.70
Communication and rapport	77.80	28.60	42.90	87.50	60.90	18.20
Privacy and confidentiality	66.70	28.60	28.60	87.50	73.90	45.50
Pain management	55.60	28.60	28.60	87.50	73.90	45.50
Timeliness	55.60	28.60	42.90	62.50	56.50	72.70
Person-centredness	66.70	57.10	42.90	50.00	43.50	54.50
Autonomy	44.40	28.60	14.30	12.50	26.10	45.50
Emotional and social support	33.30	85.70	85.70	62.50	30.40	27.30

Proportion of individual studies within life stages: 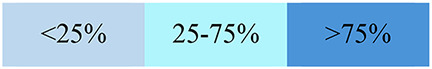

## Discussion

This review aimed to examine and describe key domains of PREMs from existing review publications on patient-reported experience of care covering distinct healthcare technical areas and life stages. We found 52 articles that presented evidence of measurement of various PREMs domains. The findings of the included literature provide an appreciable overview of PREMs across various healthcare programme technical areas and life stages. We also highlight the gaps in literature as well as future opportunities for programmatic strategy.

### PREMs domains

#### Cross-cutting domains

Our review found certain domains to cut across technical healthcare areas. Respect and dignity appeared consistently in all technical areas; similarly, communication and rapport appeared consistently. The ubiquity of these domains reflects the globally acknowledged need for the provision of dignified healthcare as a basic human right.^28^ This also corroborates evidence from prior global reviews that have highlighted the importance of communication and rapport between patients and providers, as well as the critical role communication and rapport play in quality healthcare provision.^29 30^ Of note, although communication and rapport appear consistently and are fundamental to patient experience, they are often the domains with the greatest gaps in health systems globally.^31 32^ Research into the factors driving these gaps and targeted efforts to address them are therefore needed.

#### Distinct domains

Some PREMs domains are more accentuated in certain healthcare technical areas. Pain management appeared in all paediatric studies and highlights general concern about symptom management and pain alleviation among younger patients, who generally possess lower pain thresholds.^33 34^ Pain management also appears as a domain in studies on acute and emergency care. Themes of negative patient-reported experiences in this technical area have emerged in literature, with studies continually showing discrepancies between provider and patient perceptions of pain which often translate to negative patient experiences.^35 36^ Negative experiences with pain management as reported by acute and emergency care users evidently need to be prioritised in order to effectively improve patient experiences and overall QoC delivery.

Similarly, emotional and social support as a domain was distinct in the areas of paediatric care as well as geriatric care. Individuals in these stages, inherently due to age and/or infirmity, extensively require emotional and social support from caregivers and/or providers. This is reflected in the number of studies in these stages which also included caregiver and provider perspectives.

#### Technical areas

This review discovered a disproportionately higher prevalence of PREMs literature in some technical areas from our framework. Studies on PREMs were most common in SRH programmes, as shown in table 2. The ubiquity of PREMs studies in this area, particularly within the pregnancy, birth and postnatal period, may be linked to recently concerted efforts to improve QoC across SRH programmes globally. Indeed, we are inclined to hypothesise that the proliferation of PREMs literature in this technical scope may be partially driven by recommendations from the development and implementation of WHO’s QoC framework for maternal and newborn health programmes. We further draw links between this and WHO’s multicountry network of programmes for improving QoC of maternal and newborn health.^37^ The QoC Network has garnered widespread support from its constituent and affiliate programmes.^38 39^ This is manifest in the assimilation of recommendations for prioritising measurement and evaluation of QoC components in MNCH healthcare programmes.

In contrast to SRH, technical areas such as acute and emergency care, disability care and mental health rarely appeared in PREMs reviews. This paucity of PREMs literature highlights critical gaps in the evidence necessary to systematically improve QoC in those programme themes. Literature gaps in emergency care have been previously highlighted by Obermeyer and colleagues in their review of studies from 59 LMICs.^40^ The authors resultantly recommended increased research on improving emergency healthcare delivery. Importantly, under-representation of PREMs literature in mental health and disability programmes is notably worrying, given the evidence of bias and discrimination in healthcare settings encountered by individuals with mental health conditions and disabilities. Indeed, recommendations for increased research on QoC in mental health programmes have been made by Pincus and colleagues in their evaluation of the ‘*Crossing the Quality Chasm: Adaptation to Mental Health’* campaign, citing sparse scientific literature in this technical area.^41^

#### Life stages

Our review framework attempted to examine PREMs domains by distinct life stages, as illustrated in table 3. However, we discovered that several articles in our review often investigated a single domain, across multiple populations, at various stages of the life course. This may be attributable to the all-encompassing nature of comprehensive reviews. Our analyses and synthesis, therefore, attempted as much as possible to tease out domains at discrete life stages. A technical area where life stage overlap recurrently occurred was SRH. Numerous publications examined PREMs domains across the stages of pregnancy and birth and postnatal in a single review. Similarly, studies on adulthood often operationalised adulthood as ages above 18 years, overlapping with elderly populations in our framework. A few studies, however, specifically defined their population of focus as ageing/elderly. This was not surprising in the technical area of geriatric healthcare. This clustering of several life stages within a single PREMs study elicits a challenge in distinguishing specific programme user needs at nuanced stages of life such as early adulthood, mid-life and ageing/elderly stages.

#### Time trends of PREMs studies

In our temporal analyses, we used an open-ended time search of articles published historically up to the time the review was conducted. Almost all of the literature included in our review was from the past decade, with almost half being published in the past 5 years. This may be indicative of the recent traction that PREMs have gained in healthcare programme QoC improvement efforts. This manifested especially in LMICs where exponential growth occurred in the past 5 years. Recent advancements in programmatic technology adaptation may also have facilitated the development and implementation of digital PREMs platforms, precipitating the efficient collection, analyses and utilisation of PREMs data.^2 42^ Some PREMs tools, such as the ‘*Consumer Quality Index Palliative Care questionnaire for patients’*, also appeared to undergo modification as time progressed, indicating the execution of important updates to existing measures.

#### Distribution of PREMs studies in income-specific contexts

Our analyses on the distribution of PREMs literature in countries by economic classification highlighted a relatively low prevalence in LMICs. This disparity in LMICs underscores persistent, yet important implementation gaps in comprehensively understanding and addressing QoC needs in lower-resourced contexts.^40 43^ This implementation barrier is problematic as lower resourced settings already face immense structural and systemic drawbacks in achieving programmatic QoC targets. Noteworthily, the dominant increase in the proportion of studies published in LMICs compared with HICs over the past decade suggests an increasing prioritisation of QoC in LMIC settings. Notably, gaps in literature from LMICs are also more accentuated in specific technical areas. A majority of the PREMs literature from LMICs focused on SRH; none focused on geriatric care programmes, highlighting an evidence gap in LMIC literature on ageing and elderly populations (as operationalised in our framework). This scarcity of PREMs evidence on programmes for ageing populations in LMICs is worrying, as life expectancy continues to increase, and the population of elderly individuals needing respectful, person-centred care is expanding in these contexts.^44^

#### Characteristics of included PREMs studies

Although nearly half of the reviews in this study incorporated articles from both grey and peer-reviewed literature, the majority focused only on peer-reviewed sources of literature. This could potentially omit important PREMs products, including programme reports and policy briefs that may not be available in peer-reviewed spaces but are of crucial programmatic and policy significance.^43 45^

Our observation also was that a considerable number of reviews included a mix of qualitative and quantitative studies. This we deem beneficial in providing not just a numerical summary of PREMs instruments but also presents a valuable opportunity for in-depth insights into the patients’ lived experience. This mixed-methods approach promotes a comprehensive understanding of PREMs, especially in highlighting more nuanced domains that may be obscured in purely quantitative investigations.

#### Validation and standardisation of PREMs tools

We interpreted the presence of both validated and non-validated tools in a large proportion of the included articles as evidence of the expansion and diversity of available PREMs instruments in QoC efforts. Validated measures in the context of our review are defined as having undergone a process of testing for their scientific ability to accurately and reliably measure a construct. We also considered measures that have been tested for consistency, stability and applicability to the population for which they are intended. With this in mind, we observed that a relatively small proportion of studies reported using only validated tools while almost one-third made no explicit mention of the validity of PREMs measures used. We also observed a wide variance of PREMs tools across technical programme areas and life stages and interpreted this as a challenge with harmonisation of PREMs tools in discrete categories. Validated PREMs measures are a scientific crux of generating evidence-informed programme and policy action.

### Strengths and limitations

A primary strength of this review is the application of an inclusive, comprehensive search strategy encompassing peer-reviewed and grey literature databases, as well as hand-searched recommended literature from programme and research experts in the field. Furthermore, the development of our search strategy, based on existing evidence-based WHO frameworks across healthcare technical areas and life stages, enhanced the precision and relevance of the search, ensuring alignment with established frameworks and their respective recommendations. Additionally, the iterative and consultative methodology applied, involving continuous engagement with the experts within *LSQM TWG-TT3*, extensively refined the review while enhancing our capture of non-readily available sources of literature and technical products that might have been otherwise inadvertently excluded.

Nonetheless, this review is subject to some limitations. Efforts to mitigate publication bias, including conducting manual searches of programme products recommended from the consultations, may have missed some obscure grey material. Furthermore, attribution of literature to discrete technical areas and life stages was complex and subjective to interpretation at a high level. This is because of extensive heterogeneity in several publications which reference multiple programme technical areas and life stages. Life stages as defined include some overlaps, with some broader categories, such as adulthood, masking some relevant experience of subgroups contained therein. Further, PREMs that use the broad construct of person-centredness include items that cut across several domains, including care continuity, which were difficult to tease out from the articles, thus some of the domains may be underrepresented because they are captured under broad person-centred PREMs. Finally, our review ended in 2023, thus excluding more recent publications. We, however, intend for this initial review to serve as a basis for future reviews that should focus on only the recent literature.

## Conclusions

This review found 50 review articles, one research note and one independent article in the current global landscape reporting on multifaceted PREMs instruments across the life course in various healthcare technical areas. Most of these are from HICs, although trends reveal an increasing proportion of material from LMICs. This highlights disparities in application of PREMs geographically and across technical areas and domains, which sets the stage for future priority-setting in programme QoC design and implementation. Most PREMs tools overlap in terms of technical area and life stage. This has implications for tool development aimed at comprehensive coverage of the complete life course. A generic set of PREMs measures, applicable across the entire life course, complemented by supplementary technical area-specific and stage-specific PREMs, may be beneficial in capturing common and distinct domains of patient experience of care across life stages and healthcare technical areas. We therefore recommend investments in the validation and psychometric development of PREMs measures within programmes in LMICs, with harmonisation of PREMs measures across technical areas, domains and life course stages in easily adaptable formats for global programmatic use.

## Supplementary material

10.1136/bmjopen-2025-103782 online supplemental file 1

## Data Availability

Data sharing not applicable as no datasets generated and/or analysed for this study.
