# Supplementary material for: Global scoping review of key domains of patient-reported experience of care measures across life stages and healthcare technical areas
Source: BMJ Open. 2026 Jan 16;16(1):e103782. doi: 10.1136/bmjopen-2025-103782 (PMC12815105; doi:10.1136/bmjopen-2025-103782)
Supplement: online supplemental file 1 [file bmjopen-16-1-s001.docx]

**APPENDICES**

**Appendix A: Search Terms & Strategy: Patient Reported Experience Measures (PREMS)**

We used iterations of combined medical subject headings (MeSH) in groups (Key term x Technical domain x Life stage) as presented in this appendix table to search both peer-reviewed and grey literature databases. Recommendations of literature from the *LSQM TWG* had to match these categories of search terms to be included in the review.

| **KEY TERM** | **VARIABLE TERM GROUP A (TECHNICAL DOMAINS)** | **VARIABLE TERM GROUP B (LIFE STAGE)** |
| --- | --- | --- |
| Patient Reported Experience Measures  Patient Reported Measures | Child Health  Chronic Disease  Contraception  Disability  Emergency  Family Planning  Fertility  General Outpatient Care  Geriatric  HIV/AIDS  Immunization  Maternal Health  Mental Health  Palliative  Paediatric  Sexual Reproductive Health | Pre-pregnancy [Antenatal, ANC]  Pregnancy [Gestation]  Birth [Labour, Childbirth, Parturition]  Neonatal [Newborn]  Infancy  Childhood [Toddlerhood]  Adolescence [Teenage]  Adulthood  Ageing [Elderly, Geriatric] |

1. Patient Reported Experience Measures
2. Patient Reported Measures
3. Review
4. Child Health
5. Chronic Disease
6. Contracep*
7. Disab*
8. Emergency
9. Family Planning
10. Fertility
11. General Outpatient Care
12. Geriatric
13. HIV/AIDS
14. Immunis*
15. Maternal Health
16. Mental Health
17. Palliative
18. Paediatric
19. Sexual Reproductive Health
20. 1 AND 4 OR 5 6 7 8 9 10 11 12 13 14 15 16 17 18 19 AND 3
21. 2 AND 4 OR 5 6 7 8 9 10 11 12 13 14 15 16 17 18 19 AND 3
22. Pre-pregnancy [Antenatal, ANC]
23. Pregnancy [Gestation]
24. Birth [Labour, Childbirth, Parturition]
25. Neonatal [Newborn]
26. Infan*
27. Childhood [Toddlerhood]
28. Adolesce* [Teenage*]
29. Adult*
30. Ageing [Elder*, Geriatric]
31. 1 AND 22 OR 23 24 25 26 27 28 29 AND 3
32. 2 AND 22 OR 23 24 25 26 27 28 29 AND 3

**Appendix B: PREMs Tools and Instruments Referenced in Included Publications**

1. Adult Primary Care Assessment Tool (short and expanded versions)
2. Ambulatory Care Experiences Survey (ACES)
3. Assessment of Quality of Life (AQoL) instrument
4. Breast cancer patient satisfaction with follow-up in primary care versus specialist care survey
5. CAHPS Adult Primary Care Questionnaire 1.0: Clinician and Group Survey
6. CAHPS American Indian Survey
7. CAHPS Hospice Survey
8. CAHPS Cancer Care Survey
9. Canadian Community Health Survey (CCHS)
10. Canadian Health Care Evaluation Project (CANHELP) – Bereavement/ Caregiver
11. CANHELP Patient
12. Client Perceptions of Coordination Questionnaire (CPCQ)
13. consideRATE 2021
14. Consumer Assessment of Healthcare Providers and Systems (CAHPS) Cancer Care Survey
15. Consumer Quality Index Continuum of Care
16. Consumer Quality Index GP care
17. Consumer Quality Index Palliative Care questionnaire for patients, 2012, 2013
18. Consumer Quality Index Palliative Care questionnaire for relatives, 2013
19. Consumer Satisfaction with Public Health Care Survey
20. Consultation Satisfaction Questionnaire (CSQ)
21. Duke Health Profile (the DUKE)
22. Dying Care Outcome and Process Scale Before and After Death, 2019
23. End-of-Life in Dementia Satisfaction With Care (SWC-EOLD)
24. EUROPEP
25. European Health Interview Survey
26. EuroQol EQ-5D Health Questionnaire
27. Eurocommunication Patient Questionnaire
28. Expectancies list from Nijmegen
29. FAMCARE-2
30. FAMCARE-Patient
31. Family Assessment of Treatment at the End-of-Life (FATE)
32. Family Evaluation of Hospice Care (FEHC)
33. Family Evaluation of Palliative Care (FEPC)
34. Family Satisfaction with Care in the Intensive Care Unit: FS-ICU 24R
35. Feeling Heard and Understood, 2015
36. General Practice Assessment Questionnaire (GPAQ)
37. A modified version of the General Practitioner Assessment Survey (GPAS)
38. Health Care Quality Survey (Commonwealth Fund, different versions)
39. Health Care Satisfaction Questionnaire (HCSQ)
40. Health-Care, Self-Determination Theory Packet
41. HTPN Patient Satisfaction Survey
42. Improving Practice Questionnaire (IPQ)
43. International Health Policy Survey (Commonwealth Fund, different versions)
44. Kaiser Permanente Survey
45. Medical Interview Satisfaction Scale (MISS)
46. 4-item Motherhood Discouraged Scale
47. National Survey of NHS Patients: General Practice
48. Nurse Practitioner Satisfaction Survey (NPSS)
49. Out-of-Hours Patient Questionnaire
50. Otani et al. 2020 study developed questionnaire
51. Palliative Care Clinical Network – Palliative Care experience Survey, 2020
52. Palliative Care Experience Survey
53. Patient Assessment of Communication during Telemedicine (PACT) questionnaire
54. Patient Continuity of Care Questionnaire (PCCQ)
55. Patient Expectations Questionnaire (PEQ)
56. Patient Experiences Questionnaire for Out-of-Hours Care (PEQ-OHC)
57. Patient Participation Program Survey
58. Patient Satisfaction Consultation Questionnaire (PSCQ-7)
59. Patient Satisfaction Questionnaire, 2014
60. Patient Satisfaction Survey amongst family practice patients with diverse ethnic backgrounds
61. Patient Satisfaction Survey of primary health care (PHC) services among elderly people (60 years)
62. Patient Satisfaction Survey with Primary Care Office-Based Buprenorphine/Naloxone Treatment Survey
63. Patient Satisfaction with Medical Encounters Questionnaire
64. Patient Satisfaction with Out-of-Hours Primary Care Survey
65. Patient satisfaction with visits to family physician
66. Patient–Doctor Relationship Questionnaire (PDRQ-9)
67. Patient-Reported Physician Cultural Competence (PRPCC) score
68. Patient Experience Questionnaire (PEQ)
69. Patient Satisfaction with Primary Care Survey
70. Patients Assessment Chronic Illness Care (PACIC) Questionnaire
71. Patients Satisfaction in Resident and Attending Ambulatory Care Clinics Questionnaire
72. Person-Centered Maternity Care (PCMC) Scale
73. Person-Centered Prenatal Care (PCPC) Scale
74. Physician–Patient Questionnaire (PPQ)
75. Primary Care Assessment Survey
76. Primary Care Evaluation Tool (PCET)
77. Primary Caregivers Satisfaction Survey
78. Propensity to Seek Health Care Questionnaire
79. PMA2020
80. Quality Care Questionnaire-Palliative Care (QCQ-PC), 2018
81. Quality from the Patient’s Perspective (QPP-PC), 2015
82. Quality of Communication Questionnaire (QOC), 2006
83. Quality of Death and Dying Questionnaire (QODD), 2002
84. Quality of End-of-life Care (QOELC) Survey – Family, 2010
85. Quality of End-of-life Care (QOELC) Survey – Patient, 2010
86. Quality of Visit to Family Physician Questionnaire
87. Quality of End-of-Life Care and Satisfaction with Treatment (QUEST) questionnaire, 2013
88. QUOTE for migrants
89. QUOTE
90. 3-item Restrictive Recommendations Scale
91. SF-36 (and SF-12)
92. Short Questionnaire for Out-of-Hours Care
93. Satisfaction Scale for Family Members Receiving Inpatient Palliative Care (SFIPC), 2002
94. Satisfaction with Doctors Questionnaire, 2011
95. Satisfaction with treatment decision (SWTD) survey, 2013
96. Survey of patients’ views of access to electronic health records in primary care
97. Survey of patients’ views of access to electronic health records in primary care
98. Survey of primary care patients’ preferences and their experiences with interpersonal continuity of care
99. Survey of primary care patients’ preferences and their experiences with interpersonal continuity of care
100. The '5As' model (assess, advise, agree, assist, arrange)
101. The OPTION Scale
102. The Sinclair Compassion Questionnaire
103. Victorian Palliative Care Satisfaction Instrument (VPCSI), 2016
104. Victorian Palliative Care Satisfaction Instrument, 2016
105. VOICES-SF (short form), 2019
