# Supplementary material for: Global scoping review of key domains of patient-reported experience of care measures across life stages and healthcare technical areas
Source: BMJ Open. 2026 Jan 16;16(1):e103782. doi: 10.1136/bmjopen-2025-103782 (PMC12815105; doi:10.1136/bmjopen-2025-103782)
Supplement: Supplementary file 2 [file bmjopen-2025-103782.reviewer_comments.pdf]

PEER REVIEW HISTORY

BMJ Open publishes all reviews undertaken for accepted manuscripts. Reviewers are asked to complete a checklist review form and are provided with free text boxes to elaborate on their assessment. These free text comments are reproduced below.

ARTICLE DETAILS

Title (Provisional)

A global scoping review of key domains of patient-reported experience of care measures across life stages and healthcare technical areas

Authors

Habib, Helen H H; Zhang, Connie; Hill, Kathleen; VAZ, Lara; Rawlins, Barbara; Brownwood, Ian; Tunçalp, Özge; Muzigaba, Moise; Afulani, Patience A

VERSION 1 - REVIEW

**Reviewer**	**1**
**Name**	**Askin, Nicole**
**Affiliation**	**University of Manitoba**
**Date**	**20-Aug-2025**
**COI**	**None**

Thank you for submitting this manuscript on an important topic. Unfortunately I have significant concerns with the search strategy as presented in Appendix A. This appendix does not contain a full reproducible search strategy. It also appears to be limited to use of MeSH, without any corresponding free text terms; this is inconsistent with best practices regarding scoping review search methods. I am also concerned that even among the MeSH tree, the terms used were not sufficiently broad to encompass all of the studies likely to be of interest. In short, the search appears likely to be non-comprehensive. I suggest consulting with a health sciences librarian to improve the comprehensiveness and replicability of the search strategy. I would also suggest you consider searching solely on PREMs terminology - as you noted, it is very common for a single publication to synthesize multiple areas, and therefore not all areas may be well described in indexed fields.

Some additional comments:

-The article would benefit from a thorough copyediting to ensure clarity and flow

-What do you mean by "grouped" MeSH?

-Google Scholar is not a peer-reviewed literature database

-Websites searched for grey literature are not databases

-What is the rationale for stopping the review at 2023? An update search would be warranted

-English as an inclusion criterion should be noted as a limitation

-What definition of LMIC vs HIC is being used? This should be explained and cited.

**Reviewer**	**2**
**Name**	**Gauld, Robin**
**Affiliation**	**University of Otago, Preventive and Social Medicine**
**Date**	**16-Sep-2025**
**COI**	**None**

Thank you for submitting this valuable review. The research work is very high-quality and the topic is important to be focusing on. The only point that might be useful highlighting in a bit more detail is around the question of person centredness. This is in Figure one as a key domain. The review finds that dimensions such as communication and rapport appear consistently. It may be useful coming back to this in the discussion as this is pretty fundamental to patient experience. Most health systems fail badly when it comes to being person centred. Patient experience is one of the best ways of highlighting this failure. It may be useful discussion what sort of research is needed in terms of PREMs that will bring more focus on this area. Questions around coordination from the patient perspective are fundamental to this. It may be worth touching on some of the work done in the past by The Commonwealth Fund which has looked at care coordination from a patient perspective. This work is not review work of course but rather it's focused on patient survey reporting.

**Reviewer**	**3**
**Name**	**Roemeling, Oskar**
**Affiliation**	**University of Groningen Faculty of Economics and Business**
**Date**	**22-Sep-2025**
**COI**	**None**

Peer Review of "A Global Scoping Review of Key Domains of Patient-Reported Experience of Care Measures Across Life Stages and Healthcare Areas"

Dear Editor,

Thank you for the opportunity to review the manuscript titled "A Global Scoping Review of Key Domains of Patient-Reported Experience of Care Measures Across Life Stages and Healthcare Areas" by Habib et al. The authors present a well-written and methodologically

sound review of the application of Patient-Reported Experience Measures (PREMs) across diverse domains. The manuscript is clear, and the methods are thoroughly explained.

I have a few minor comments and requests for clarification, as well as suggestions for expanding or refining the presentation of findings. My feedback is outlined below:

1. Currency of Included PREM Measures

The review includes studies up to 2023, which raises a question: Could some of the earlier PREM measures included in the analysis now be outdated or superseded by more recent versions? For example, early studies may have used preliminary versions of tools that have since evolved. I recommend the authors reflect on this potential limitation and clarify whether any included measures have been updated or replaced. Perhaps a paragraph on pg22 (under time trends)?

2. Clarification on "Technical Input"

The methods section mentions that the LSQM-TWG-TT3 team provided "technical input." Could the authors specify what this entails? For instance: What types of questions or discussions were addressed? Did this input relate to methodological guidance, tool validation, or domain-specific expertise? A brief example would help readers understand the nature of this contribution.

3. Inclusion of Individual Studies in Absence of Reviews

The authors note that individual studies were included in domains where reviews were unavailable. To improve transparency, I suggest a) specifying how many individual studies were included for this reason, and b) clarifying the criteria for these studies: Were they limited to PREM development (e.g., reporting on tool validation) or did they also include applied studies (e.g., PREMs used in improvement projects)?

4. Clarifying "Individual Studies" on Page 11

On page 11, the authors state that most included articles are "individual studies." Does this mean these are primary studies rather than reviews? If so, this distinction should be made explicit to avoid confusion.

5. Ambiguity in Reporting on Page 15

The wording on page 15 is unclear to me regarding whether the authors are discussing:

a) The reviews included in their scoping review, or

b) The individual studies cited within those reviews.

For example, the sentence "Less than a quarter of the included studies (...) only included individual studies (...)" is confusing to me. I recommend revising this section to clearly distinguish between findings from included reviews, and findings from individual studies within those reviews.

6. Expanding Table 2 for Deeper Insights

Table 2 presents a valuable overview of PREM domains, but it might be enhanced by listing the top 3 most commonly applied PREMs per domain to highlight prevalent tools. Moreover, incorporating a temporal analysis would increase insights even further: Has the use of certain PREMs evolved over time? For example, are newer measures replacing older ones, or are specific domains seeing shifts in preferred tools?

Overall, this is an insightful review and the suggestions above aim to enhance the value of the work, I hope the authors will find these useful. Thank you for the opportunity to review this work.

VERSION 1 - AUTHOR RESPONSE

Reviewer:1

Ms. Nicole Askin, University of Manitoba

Comments to the Author:

Thank you for submitting this manuscript on an important topic. Unfortunately I have significant concerns with the search strategy as presented in Appendix A. This appendix does not contain a full reproducible search strategy. It also appears to be limited to use of MeSH, without any corresponding free text terms; this is inconsistent with best practices regarding scoping review search methods. I am also concerned that even among the MeSH tree, the terms used were not sufficiently broad to encompass all of the studies likely to be of interest. In short, the search appears likely to be non-comprehensive. I suggest consulting with a health sciences librarian to improve the comprehensiveness and replicability of the search strategy. I would also suggest you consider searching solely on PREMs terminology - as you noted, it is very common for a single publication to synthesize multiple areas, and therefore not all areas may be well described in indexed fields.

Response: Thank you for your helpful feedback. A sample search strategy for PubMed has been added to Appendix A to demonstrate how articles were searched using free text terms and MeSH. Additionally, the search terms have been updated to include a more comprehensive list

of the free text terms that were used in our searches. Of note, these search terms were developed with the help of a health sciences librarian

Some additional comments:

-The article would benefit from a thorough copyediting to ensure clarity and flow

Response: Thank you for your feedback. We have thoroughly copyedited the manuscript.

-What do you mean by "grouped" MeSH?

Response: The statement "*We used a combination of grouped medical subject headings (MeSH) related to life stages and healthcare technical domains*" describes the aggregation of the search terms we used under the three broad themes of life stages, technical areas & PREMS domains. This is visually presented in Appendix 1.

-Google Scholar is not a peer-reviewed literature database

Response: We have modified the language of the text to reflect that we searched Google Scholar specialized search engine

-Websites searched for grey literature are not databases

Response: We have modified the language of the text to indicate that grey literature sources were searched

-What is the rationale for stopping the review at 2023? An update search would be warranted

Response: This work was commissioned and conducted in 2023 by the LSQM TWG and include all literature up to the inception of the search. However, a considerable amount of time was spent in synthesizing all the available literature. We have noted the limitation in the timeframe of included studies in the limitations section. Given the extent of the literature already synthesized, we politely advance that it is important to publish this review providing

historical literature up to 2023 to provide a basis for future reviews that will no longer need to start from inception but will build on and update this existing work.

-English as an inclusion criterion should be noted as a limitation

Response: We have noted the language limitation of only English articles in the added limitations section

-What definition of LMIC vs HIC is being used? This should be explained and cited.

Response: Thank you; we have provided and referenced the definition of LMICs/HICs as per the the World Bank classification

We thank you for your insightful comments on our manuscript.

Reviewer: 2

Dr. Robin Gauld, University of Otago

Comments to the Author:

Thank you for submitting this valuable review. The research work is very high-quality and the topic is important to be focusing on.

The only point that might be useful highlighting in a bit more detail is around the question of person centredness. This is in Figure one as a key domain. The review finds that dimensions such as communication and rapport appear consistently. It may be useful coming back to this in the discussion as this is fundamental to patient experience. Most health systems fail badly

when it comes to being person centred. Patient experience is one of the best ways of highlighting this failure. It may be useful discussion what sort of research is needed in terms of PREMs that will bring more focus on this area. Questions around coordination from the patient perspective are fundamental to this. It may be worth touching on some of the work done in the past by The Commonwealth Fund which has looked at care coordination from a patient perspective. This work is not review work of course but rather it's focused on patient survey reporting.

Response: Thank you for your helpful feedback and input. In response to your comment, we have added a short section to the discussion to note that, although communication and rapport appear consistently and are fundamental to patient experience, they are often the domains with the greatest gaps in health systems globally. Research into the factors driving these gaps and targeted efforts to address them are therefore needed. We have also noted the limitation of including person-centeredness as a domain due to how it was presented in some of the articles, as it includes several other domains including continuity which is prominent in the work by the Commonwealth Fund. We also mention the Picker Patient Survey as one of the most frequently cited validated PREMS measure, which we understand is part of the work by the Commonwealth Fund, which helped establish the Picker Institute as a separate organization to advance the principles of patient-centred care following a merger of the Picker Foundation and the Commonwealth Fund to create the Picker/Commonwealth Patient-Centred Care Program. Of note, however, there is a growing body of literature on interventions addressing these gaps, however, we reckon it will be going beyond the scope of this manuscript if we delve into additional detail on this.

We thank you for your insightful comments on our manuscript.

Reviewer: 3

Dr. Oskar Roemeling, University of Groningen Faculty of Economics and Business

Comments to the Author:

Dear Editor,

Peer Review of "A Global Scoping Review of Key Domains of Patient-Reported Experience of Care Measures Across Life Stages and Healthcare Areas"

Thank you for the opportunity to review the manuscript titled "A Global Scoping Review of Key Domains of Patient-Reported Experience of Care Measures Across Life Stages and Healthcare Areas" by Habib et al. The authors present a well-written and methodologically sound review of the application of Patient-Reported Experience Measures (PREMs) across diverse domains. The manuscript is clear, and the methods are thoroughly explained. I have a few minor comments and requests for clarification, as well as suggestions for expanding or refining the presentation of findings. My feedback is outlined below:

1. Currency of Included PREM Measures

The review includes studies up to 2023, which raises a question: Could some of the earlier PREM measures included in the analysis now be outdated or superseded by more recent versions? For example, early studies may have used preliminary versions of tools that have since evolved. I recommend the authors reflect on this potential limitation and clarify whether any included measures have been updated or replaced. Perhaps a paragraph on pg22 (under time trends)?

Response: Thank you for your helpful feedback. A section has been added to the discussion section noting the update of some PREMS tools to newer versions in more recent studies. It

has also been noted in the “Limitations” section of the study that searches were conducted until 2023 and thus this study may be missing any material published after that time period. We anticipate that this review will serve as a basis for future updated reviews that build on the existing evidence up to 2023 to focus on only more recent work.

2. Clarification on "Technical Input"

The methods section mentions that the LSQM-TWG-TT3 team provided "technical input." Could the authors specify what this entails? For instance: What types of questions or discussions were addressed? Did this input relate to methodological guidance, tool validation, or domain-specific expertise? A brief example would help readers understand the nature of this contribution

Response: Thank you for your helpful feedback. The methods section of this manuscript has been updated with in-depth detail on the technical contributions of the LSQM-TWG to the conduct of the review including protocol development and literature source recommendations.

3. Inclusion of Individual Studies in Absence of Reviews

The authors note that individual studies were included in domains where reviews were unavailable. To improve transparency, I suggest a) specifying how many individual studies were included for this reason, and b) clarifying the criteria for these studies: Were they limited to PREM development (e.g., reporting on tool validation) or did they also include applied studies (e.g., PREMs used in improvement projects)?

Response: Thank you for your helpful feedback. The breakdown of review articles and single study articles has been added in the “Characteristics of included studies section”. Of note, only one individual study was included in the articles for this current review. We had also previously specified in the methodology section “...In those scenarios, individual studies that were considered eligible were those that aimed to report on a PREMs instrument for use in a

healthcare technical area or life course domain. This excluded protocols and metrics development studies”

4. Clarifying "Individual Studies" on Page 11

On page 11, the authors state that most included articles are "individual studies." Does this mean these are primary studies rather than reviews? If so, this distinction should be made explicit to avoid confusion.

Response: Thank you for your helpful guidance. The sentence has been updated to convey that the included review articles included in this work reported on individual studies from the countries mentioned. Notably, only one individual study is included in this current review. We have also clarified how we use the terms articles and individual studies in this manuscript in an operationalisation statement.

5. Ambiguity in Reporting on Page 15

The wording on page 15 is unclear to me regarding whether the authors are discussing:

a) The reviews included in their scoping review, or

b) The individual studies cited within those reviews.

For example, the sentence "Less than a quarter of the included studies (...) only included individual studies (...)" is confusing to me. I recommend revising this section to clearly distinguish between findings from included reviews, and findings from individual studies within those reviews.

Response: Thank you for your helpful guidance. The article has been proofread to harmonize the use of the terms “article” and “included/individual studies”. An operationalisation statement has been added to indicate that “article” refers to the retrieved articles in this

review whilst “included/individual studies” refers to the aggregated studies within the retrieved review articles

6. Expanding Table 2 for Deeper Insights

Table 2 presents a valuable overview of PREM domains, but it might be enhanced by listing the top 3 most commonly applied PREMs per domain to highlight prevalent tools.

Moreover, incorporating a temporal analysis would increase insights even further: Has the use of certain PREMs evolved over time? For example, are newer measures replacing older ones, or are specific domains seeing shifts in preferred tools?

Response: Thank you for this important observation. We believe that Table 3: Matrix of PREMs domains by life stages partially addresses this suggestion of highlighting the most commonly applied PREMs domains. However, given that the focus of this review was highlighting PREMs domains and not specific PREMS instruments, we think this is beyond the scope of the current review to include most common instruments for each domain. Furthermore, many of the instruments cover several overlapping domains (as we have already highlighted in the review), which will likely make such a list duplicative. We have also added observations of the version updates of the PREMs tools in the “Time Trends” section. Overall, this is an insightful review and the suggestions above aim to enhance the value of the work, I hope the authors will find these useful. Thank you for the opportunity to review this work.

Response: We thank you for your insightful comments on our manuscript.

Reviewer: 1 competing interests.: Not applicable

Reviewer: 2 competing interests.: na

Reviewer: 3 competing interests.: Not applicable

VERSION 2 - REVIEW

Reviewer 3

Name Roemeling, Oskar

Affiliation University of Groningen Faculty of Economics and Business

Date 21-Nov-2025

COI

Dear editor, the authors have adequately responded to my earlier comments. I have no further feedback for this manuscript.
